# Running for Exercise Mitigates Age-Related Deterioration of Walking Economy

**DOI:** 10.1371/journal.pone.0113471

**Published:** 2014-11-20

**Authors:** Justus D. Ortega, Owen N. Beck, Jaclyn M. Roby, Aria L. Turney, Rodger Kram

**Affiliations:** 1 Department of Kinesiology & Recreation Administration, Humboldt State University, Arcata, California, United States of America; 2 Department of Integrative Physiology, University of Colorado, Boulder, Colorado, United States of America; Scientific Institute Foundation Santa Lucia, Italy

## Abstract

**Introduction:**

Impaired walking performance is a key predictor of morbidity among older adults. A distinctive characteristic of impaired walking performance among older adults is a greater metabolic cost (worse economy) compared to young adults. However, older adults who consistently run have been shown to retain a similar running economy as young runners. Unfortunately, those running studies did not measure the metabolic cost of walking. Thus, it is unclear if running exercise can prevent the deterioration of walking economy.

**Purpose:**

To determine if and how regular walking vs. running exercise affects the economy of locomotion in older adults.

**Methods:**

15 older adults (69±3 years) who walk ≥30 min, 3x/week for exercise, “walkers” and 15 older adults (69±5 years) who run ≥30 min, 3x/week, “runners” walked on a force-instrumented treadmill at three speeds (0.75, 1.25, and 1.75 m/s). We determined walking economy using expired gas analysis and walking mechanics via ground reaction forces during the last 2 minutes of each 5 minute trial. We compared walking economy between the two groups and to non-aerobically trained young and older adults from a prior study.

**Results:**

Older runners had a 7–10% better walking economy than older walkers over the range of speeds tested (p = .016) and had walking economy similar to young sedentary adults over a similar range of speeds (p = .237). We found no substantial biomechanical differences between older walkers and runners. In contrast to older runners, older walkers had similar walking economy as older sedentary adults (p = .461) and ∼26% worse walking economy than young adults (p<.0001).

**Conclusion:**

Running mitigates the age-related deterioration of walking economy whereas walking for exercise appears to have minimal effect on the age-related deterioration in walking economy.

## Introduction

Walking performance typically deteriorates with advanced age [1], and impaired walking performance is a key predictor of morbidity among older adults [2]. A distinctive characteristic of impaired walking performance among older adults is a 15–20% greater metabolic cost for walking (worse economy) compared to young adults [3]–[5]. Several factors are known to determine the metabolic cost of walking in humans across all ages. These major biomechanical factors include the costs associated with: supporting body weight, performing mechanical work, leg swing and balance [5]–[8]. Studies investigating age-related biomechanical determinants of walking cost have found that older adults have a similar cost of balance and perform a similar amount, or even less, external mechanical work during walking as young adults [5], [9], [10]. Despite these similarities, other studies suggest that a decrease in muscular efficiency and an increase in antagonist leg muscle co-activation, contribute to the greater cost of walking in both healthy sedentary and active older adults [3], [5], [7], [10], [11]. Yet, no study has found a sole mechanical determinant that accounts for the 15–20% greater metabolic cost of walking in older adults. Therefore, interventions for improving walking economy in older age have been elusive.

Recent studies by Thomas et al. [12] and Malatesta et al. [13] show that vigorous walking interval training effectively reduces the metabolic cost of walking in older adults by as much as 20%. Yet, the mechanisms for the decreases were not elucidated. Conversely, a generalized year-long training program that included resistance, aerobic and balance exercises had no effect on post-training walking economy in older adults [14]. The different effects of these exercise interventions, high intensity aerobic versus generalized exercise with only a moderate aerobic component, suggest higher intensity aerobic activities may mitigate the typical age-related decrease in walking economy, and consequently, preserve mobility into older age.

In contrast, running economy does not exhibit the same age-related trend as walking economy. Two studies have reported that adults (45–61 years) who consistently participated in running exercise retain a similar metabolic economy of running as young runners (23–27 years) [15], [16]. Although these results seem to support the hypothesis that vigorous aerobic exercise mitigates the decline in locomotion economy, i.e. metabolic cost of running and walking, it is also possible that a decline in running economy does not occur until late into the 6^th^ decade of life, as observed with walking economy [17]. Perhaps the subjects in these studies [15], [16] were not “old” enough to exhibit declines in locomotion economy. Another possible explanation is that running economy, unlike walking economy, is simply not affected by age. However, since these running studies did not measure walking economy, it remains unclear if regular participation in running exercise mitigates the typical age-related deterioration of walking economy.

Our purpose was to determine if and how regular participation in walking or running exercise affects the metabolic cost and biomechanics of walking in older adults. We hypothesized that older runners would consume less metabolic energy for walking than older walkers. Further, we also investigated whether the two groups demonstrate different walking biomechanics. We measured metabolic rates, ground reaction forces and spatio-temporal stride variables of two groups, older walkers and older runners, while they walked on a dual-belt, force-sensing treadmill at three speeds.

## Methods

### Subjects

Thirty healthy older adults (15 males and 15 female) who either walk (4 Male, 11 Female) or run (10 Male, 5 Female) regularly for exercise volunteered. Table 1 summarizes the anthropometric characteristics of the subjects. We recruited subjects with a minimum age of 65 years, which is in accordance with prior studies reporting age-related impairments of walking performance become most apparent at this age [3], [18]–[20]. All subjects were free of neurological, orthopedic and cardiovascular disorders. Walkers self-reported walking for exercise three or more times per week for at least 30 minutes per bout and for at least six months prior to the study. Runners self-reported running for exercise three or more times per week for at least 30 minutes per bout and for at least six months prior to the study. The experiment was performed in accordance with the ethical standards of the 1964 Declaration of Helsinki and was approved by the Humboldt State University and University of Colorado Institutional Review Boards. All subjects gave written informed consent prior to participation in the study.

**Table 1:** Subject characteristics (Mean ±SD) with statistics for older walkers and older runners.

	Older Walkers (n = 15; 4M, 11 F)	Older Runners (n = 15; 10M, 5 F)
Age, years	68.9±3.0	68.9±4.7
Height, m	1.61±0.09	1.70±0.09*
Leg length, m	0.83±0.06	0.88±0.06
Body mass, kg	61.7±11.0	66.5± 13.0
Lean tissue mass, kg	39.2±7.1	48.6±9.2*
Body fat, % body mass	31.5±9.6	23.4±6.0*
*V*O_2_ Max, mlO_2_/kg/min	27.7±3.6	37.3±5.3*
Standing metabolic rate, W/kg	1.34±0.21	1.26±0.14
0.75 m/s, gross metabolic power, W/kg	3.39±0.33	3.18±0.31*
1.25 m/s, gross metabolic power, W/kg	4.33±0.56	3.97±0.40*
1.75 m/s, gross metabolic power, W/kg	6.33±0.71	5.95±0.52*

Asterisk indicates the only significant group difference (p<.05).

### Protocol

Subjects completed three sessions. In the first session, subjects underwent a physician's examination to determine neurological, orthopedic and cardiovascular health, a body composition test (DXA) to determine percent body fat and lean tissue mass and a *VO_2_* max treadmill test to determine maximal aerobic capacity. In the second session, at least five days following the first session, we measured standing metabolic rate and familiarized the subjects to treadmill walking. For the treadmill familiarization, subjects walked on a dual-belt, force-instrumented treadmill (FIT, Bertec Corporation, Columbus, OH, USA) at three speeds (0.75, 1.25 and 1.75 m/s) for at least 7 minutes at each speed. These speeds correspond to 1.67, 2.80, 3.91 MPH. Thus, subjects completed a minimum of 21 minutes total of walking familiarization. This familiarization period is over double the recommended minimum treadmill habituation time of 10 minutes [21], [22]. In the third session, at least two days following familiarization, we measured each subject's metabolic rate during quiet standing and while walking on the treadmill at three speeds (0.75, 1.25 and 1.75 m/s) in random order. All trials were five minutes in duration with at least five minutes of rest between trials. Throughout each trial, we measured the rates of oxygen consumption (*VO_2_*) and carbon dioxide production (*VCO_2_*) in order to determine metabolic rate. We calculated the average *V*O*_2_* and *VC*O*_2_* for the last two minutes of each trial. We also measured ground reaction forces (GRFs) from the force-instrumented treadmill for 1 minute during the last 2.5 minutes of each trial to determine kinetics and spatio-temporal stride variables.

### Metabolic Power Consumption

We measured *VO_2_* and *VCO_2_* using an open-circuit expired gas analysis system (TrueOne 2400, ParvoMedic, Sandy, UT, USA). We calculated average gross metabolic power per kilogram body mass (W/kg) [23] using the average *VO_2_* (mlO_2_/min) and *VCO_2_* (mlCO_2_/min) for the last two minutes of each trial, when *VO_2_* and respiratory exchange ratio reached steady state ensuring that each subject was working sub-maximally and oxidative metabolism was the main metabolic pathway. We then divided gross metabolic power by speed to calculate gross metabolic cost of transport (CoT) (J/kg/m) for walking.

### Ground Reaction Forces and Spatio-temporal Stride Variables

For each walking trial, we collected the ground reaction forces (vertical and horizontal components) of each leg from the force-sensing treadmill at 2000 Hz for a 1 minute period during the last 2.5 minutes of each trial. A custom MATLAB script (Math Work Inc., Natick, Mass) was then used to process all force data. The GRF data were filtered with a 4^th^ order zero-lag low pass Butterworth filter with a cutoff frequency of 30 Hz. For each trial, we calculated vertical and horizontal peak GRFs across all 10 strides. Using the filtered GRF data, we determined gait cycle events and spatio-temporal stride variables (stride frequency, stance time, and duty factor as percent of the gait cycle) for 10 strides of each trial (10 steps per each leg).

### Statistical Analyses

We used a repeated-measures ANOVA (p<.05) to determine statistical differences due to exercise group (walkers vs. runners) and walking speed, as well as, the exercise group-walking speed interaction. When a significant main effect of exercise group was found, we performed independent-samples t-tests with Bonferroni correction to determine at which speed(s) the differences occurred. To determine if difference in metabolic cost and GRF was related to sex differences in our runner and walker groups, we examined differences in metabolic cost, ground reaction forces and spatio-temporal stride variables due to sex among each group and analyzed difference in metabolic cost, GRFs and spatio-temproal stride variables using sex as a covariate. We found no effect of sex on any dependent variable and differences between runners and walkers were not affected by sex. We performed all statistical analyses using SPSS 21.0 (SPSS, Inc.) software. In addition to our comparison between older walkers and runners, we used a mixed-model repeated-measures ANOVA (p<.05) to make further post-hoc comparisons of gross metabolic cost in walkers and runners collected in the present study to data for young and older sedentary adults previously collected in our lab at similar speeds [5]. To make these comparison between exercise/age group (old walkers, old runners, old sedentary and young sedentary) using a linear mixed model, walking speed squared (m/s)^2^ was used as the repeated measure.

## Results

**Figure 1:**
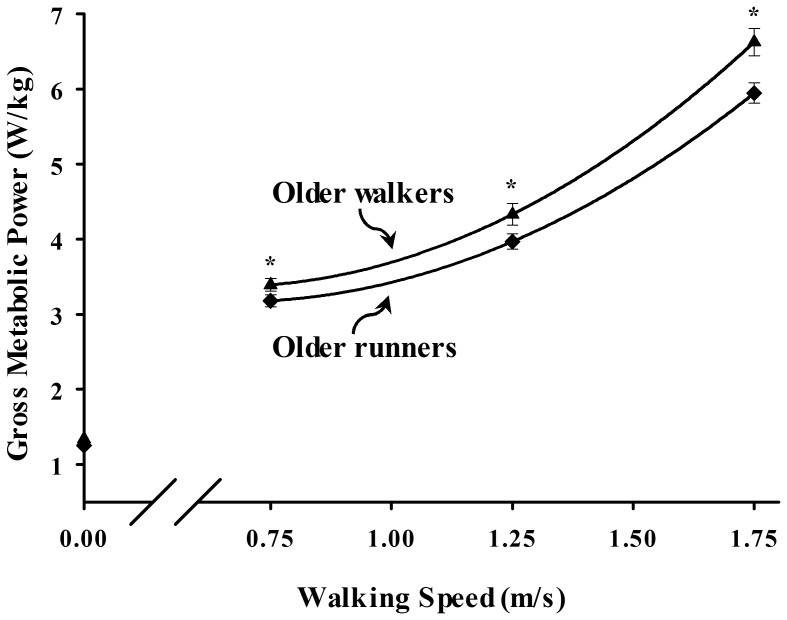
Mean (SE) gross metabolic power as a function of walking speed in older walkers (▴) and older runners (⧫) walkers (▴). Lines represent least square regression for older walkers (y = 2.709x^2^–3.539x+4.523, r^2^ = 0.86) and older runners (y = 2.382x^2^–3.189x+4.233, r^2^ = 0.89). Symbols shown on vertical axis represent standing metabolic rate of both groups. Asterisks (*) indicate significant differences between older runners and walkers (p<0.05).

In support of our hypothesis, older runners consumed 7–10% less metabolic energy for walking than older walkers across the range of speeds tested (Fig. 1; p = .016). Gross metabolic power consumption increased significantly across the range of walking speeds tested in both older runners and walkers, (p<.0001). Compared to walking at the slowest speed of 0.75 m/s, gross metabolic power increased by 95% to walk at 1.75 m/s in older walkers but only 86% in older runners (speed X group interaction, p = .009). Mass-specific standing metabolic rates were similar between older runners and walkers (p = .250; Table 1).

**Figure 2:**
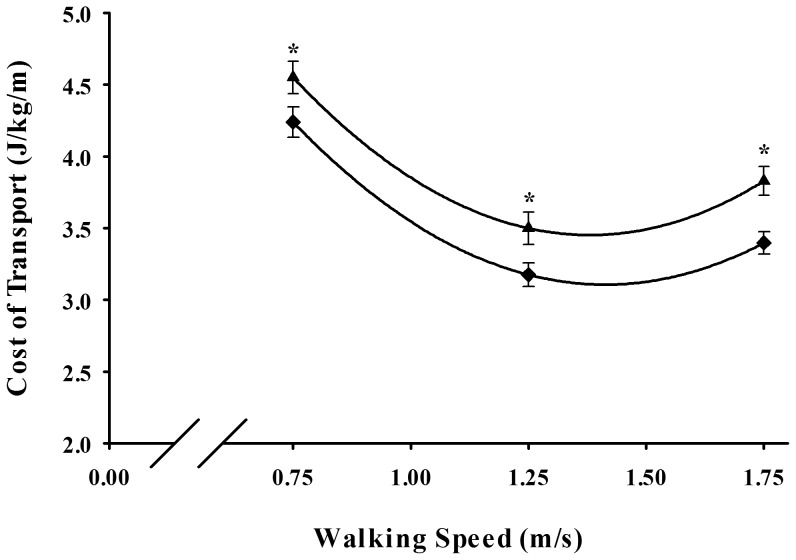
Mean (SE) gross metabolic cost of transport as a function of speed in older walkers (▴) and older runners (⧫). Asterisks (*) indicate significant differences between older walkers and runners (p<.05).

Following from the metabolic rate data, the older runners had an average of 7–10% lower gross metabolic cost of transport compared to the older walkers. Older walkers and runners exhibited similar U-shape relations between gross CoT and walking speed (Fig. 2). Between the three speeds, gross CoT was significantly lower at the intermediate speed of 1.25 m/s as compared to the faster and slower walking speeds in both the older walkers (3.49±0.09 J/kg/m, p<.0001) and older runners (3.18±0.08 J/kg/m, p<.0001). Although there were a greater number of male runners in the study, our statistical analysis showed that the difference in metabolic cost between runners and walkers was not due to sex or any other anthropometric variable.

**Figure 3:**
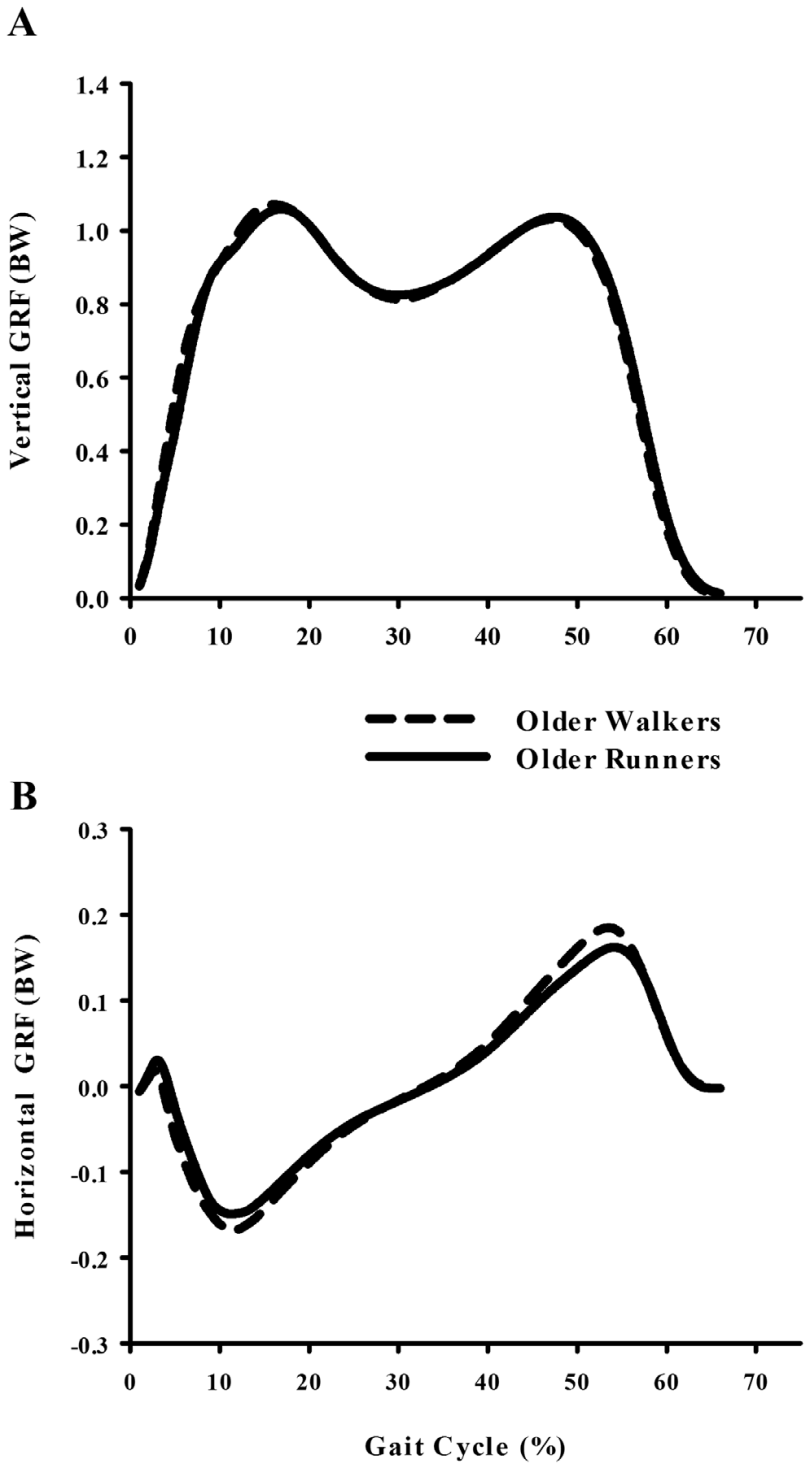
Average individual leg vertical (A) and horizontal (B) ground reaction force for older walkers (dashed lines) and older runners (solid lines) at the intermediate walking speed of 1.25 m/s.

Despite the substantial differences in walking economy, older walkers and runners exhibited nearly identical spatio-temporal stride variables and kinetics across the range of speeds (Table 2). Among spatio-temporal gait characteristics, we found no significant differences between older walkers and older runners in regards to stride time, stride frequency (p = .879), single leg stance time (p = .126) or duty factor (p = .126). However, older runners walked with slightly (6%) shorter strides in relation to their leg length compared to older walkers (p = .033). This difference remained nearly constant across the range of speeds. With regards to ground reaction forces, older walkers and runners exhibited similar first (p = .838) and second (p = .282) peak vertical ground reaction force (Figure 3). Additionally, peak anterior-posterior braking (p = .182) and propulsive (p = .056) ground reaction forces were similar for both exercise groups.

**Table 2:** Spatio-temporal stride variables and ground reaction force data (Mean ±SD) with statistics for older walkers and older runners.

	Older Walkers (n = 15)	Older Runners (n = 15)
**Speed 0.75 m/s**		
Stride Time, sec	1.26±0.11	1.19±0.08
Stance Time, % of stride	65±2	66±1
Swing Time, % of stride	35±2	34±1
Stride Frequency, Hz	0.80±0.07	0.84±0.06
Stride Length, Leg Length	1.14±0.08	1.02±0.10*
First Peak VGRF, BW%	104±3	104±3
Second Peak VGRF, BW%	101±3	100±2
Braking HGRF, BW%	−8±1	−8±1
Propulsive HGRF, BW%	11±2	10±1
**Speed 1.25 m/s**		
Stride Time, sec	1.04±0.07	1.05±0.06
Stance Time, % of stride	63±2	64±2
Swing Time, % of stride	37±2	37±2
Stride Frequency, Hz	0.97±0.07	0.95±0.06
Stride Length, Leg Length	1.57±0.08	1.49±0.10*
First Peak VGRF, BW%	110±5	108±4
Second Peak VGRF, BW%	106±5	105±3
Braking HGRF, BW%	−17±2	−16±2
Propulsive HGRF, BW%	19±0.02	17±2
**Speed 1.75 m/s**		
Stride Time, sec	0.92±0.05	0.93±0.04
Stance Time, % of stride	61± 1	63±2
Swing Time, % of stride	39±1	38±2
Stride Frequency, Hz	1.09±0.06	1.08±0.05
Stride Length, Leg Length	1.88±0.10	1.83±0.10
First Peak VGRF, BW%	134±12	129±4
Second Peak VGRF, BW%	110±9	119±7
Braking HGRF, BW%	−28±2	−26±5
Propulsive HGRF, BW%	26±3	25±3

Peak vertical ground reaction forces (VGRF) and horizontal ground reaction forces (HGRF) are represented as % body weight (BW). Asterisk indicates significant group difference (p<.05).

**Figure 4:**
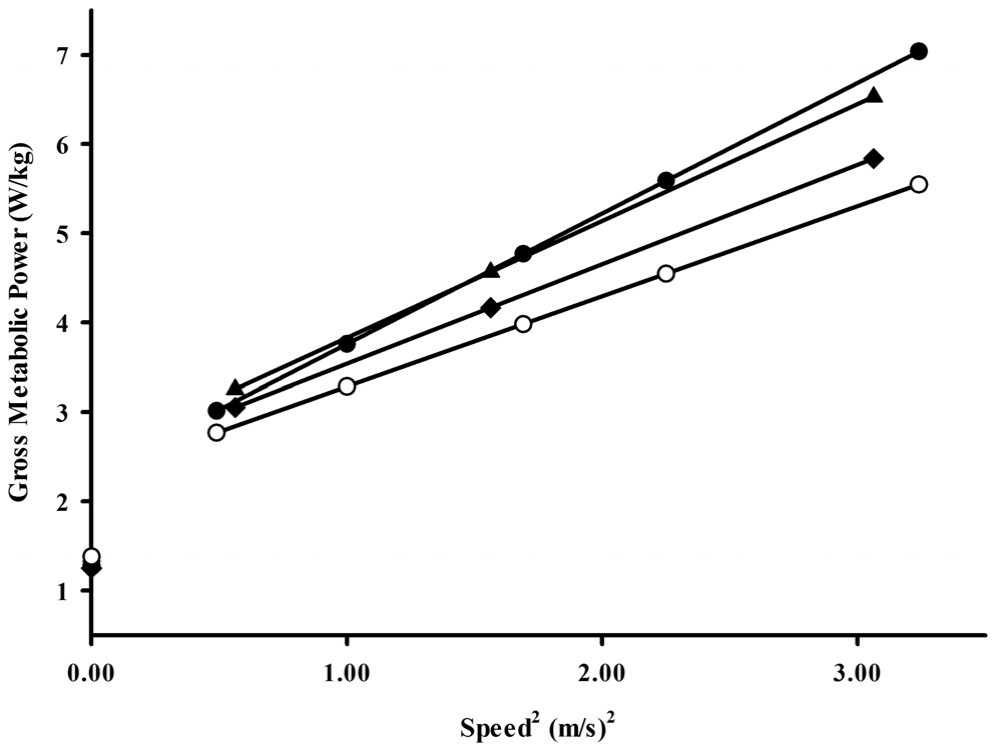
Gross metabolic power as a function of speed^2^ in older sedentary adults (•), older walkers (▴), older runners (⧫), and young sedentary adults (○). Lines denote least square regression within each group (older sedentary: y = 1.46x+2.30, r^2^ = 0.91; older walkers: y = 1.31x+2.52, r^2^ = 0.86; older runners: y = 1.12x+2.42, r^2^ = 0.88; young sedentary: y = 1.01x+2.27, r^2^ = 0.87). Symbols on vertical axis represent standing metabolic rate of each group.

We also compared gross metabolic cost of walking for older walkers and older runners to data from young and older sedentary adults collected in our lab from a prior study over a similar range of speeds [5]. The speeds used in these two studies were slightly different. Thus, in order to statistically make this comparison using a linear mixed model repeated measures ANOVA, we determined gross metabolic power as a function of speed squared (Fig. 4). The results of this analysis showed that across the range of speeds, older walkers consume metabolic energy at a similar rate as sedentary older adults (p = .461) and 14–22% faster than young sedentary adults (p<.0001). In contrast, older runners consume metabolic energy at a slower rate compared to older sedentary adults (p = .016). However, our most striking finding was that older runners consumed metabolic energy at a similar rate as young sedentary adults across the range of walking speeds (p = .237).

## Discussion and Conclusions

In this study, we distinguished the effects of regular walking vs. running exercise on the metabolic cost and biomechanics of walking in older adults. In support of our hypothesis, older runners consumed less metabolic energy for walking than older walkers. Although the older runners consumed less metabolic energy for walking than the older walkers, the two groups had almost identical walking biomechanics.

Given that there were virtually no differences in walking biomechanics between the older walkers and runners, other factors must underlie the lower cost of walking observed for the older runners. One factor may be muscle co-activation. Older adults, both sedentary and active walkers, use 30–50% greater co-activation of antagonist leg muscles compared to young adults [6], [10], [24]. It has been suggested that older adults may use greater co-activation to increase joint stiffness and the stabilization of the body, thus reducing the risk of walking related falls [25]. Yet, increased co-activation has been associated with increased metabolic cost of walking in older adults [6], [10]. It is possible that older runners are able to maintain a lower metabolic cost of walking compared to older walkers because they use less antagonist leg muscle activation. Some research shows that older adults who participated in a lower limb strength training program reduce leg muscle co-activation by 5–10% [26]. Perhaps, by regularly running three or more times per week for 30 minutes per bout, older runners are able to maintain or even increase leg muscle strength and reduce co-activation. However, a decrease in co-activation associated with running that is similar in magnitude to the decrease observed after strength training is likely not sufficient to explain the 7–10% difference in metabolic cost of walking. It is also possible that other neuromuscular factors such as widening of EMG/motoneuronal bursts [27] may also help to explain the difference in metabolic cost between older runners and walkers.

Better muscular efficiency may also help explain why older runners have a lower metabolic cost of walking than older walkers. Aging has been associated with reduced muscular efficiency [10], [28]. More specifically, mitochondrial dysfunction associated with the uncoupling of oxidative phosphorylation (reduced ATP synthesis per O_2_ uptake) effectively reduces muscular efficiency and increased the metabolic cost of muscle activation [28]. Interestingly, recent evidence suggests that aerobic exercise training may ameliorate mitochondrial uncoupling and improve muscular efficiency in older adults [29].

Perhaps studies of cycling efficiency in older adults can provide insight. In contrast to the effects of running we have observed, the muscular efficiency of cycling declines with age despite regular cycling exercise [30]. More recently, Brisswalter et al. [31] measured the cycling efficiency of active triathletes (who regularly swim, bike, and run for exercise) across age-groups and found a decline in cycling efficiency past the 5^th^ decade. These data suggest that older cyclist and triathletes are unable to maintain muscular efficiency with age. However, Peiffer et al. [32] found no difference in cycling efficiency between their youngest age group (39±3 years) and their oldest (65±4 years). Intriguingly, their oldest training group cycled 58 km more per week (359 km per week) than the youngest group. Possibly the greater quantity of aerobic cycling exercise mitigated the decrease in muscular efficiency with age.

Alternatively, the intensity of exercise may hold the key to maintaining or improving muscular efficiency. Two prior studies have found that 6–7 weeks of vigorous aerobic exercise (fast walking) that elicits a heart rate close to the ventilatory threshold can improve walking economy by 8–20% [12], [13]. More vigorous aerobic exercise such as walking uphill, fast walking or running may be required to elicit improvement in walking economy. Clinicians and others who work with older adults to improve their fitness may need to prescribe more vigorous, more prolonged and/or more frequent aerobic exercise to prevent the decline in walking performance. To test this hypothesis and help guide clinicians, a future study should investigate the effects of different intensity aerobic exercises on muscular efficiency and more specifically, the economy of walking.

### Limitations

One limitation of the current study is the cross-sectional design. It is possible that older runners may not be economical walkers because of the effect of running exercise but rather they run because they are more economical in their locomotion. To better address this issue, a future study might quantify the longitudinal effects of a running training program. One such study conducted by Trappe et al. [16] on the longitudinal effect of running exercise on running economy spanned 22 years. In that study, Trappe et al. [16] showed that running economy did not decline in older adults who maintained their health and fitness over the 22 year period, whereas runners who became unfit had worse running economy. Although these results suggest that running may help to prevent a decline in running economy, Trappe et al. [16] did not measure walking economy.

Another potential limitation of the current study is the different numbers of male and female participants in each group. Although the sex difference may have influenced the difference in anthropometrics between runners and walker, our results showed no main effect of sex on walking economy (p = .211) and no sex difference in walking economy among older runners (p = .131) or older walkers (p = .331). Based on post-hoc power analysis, it is clear that we did not have sufficient statistical power to detect sex differences that might exist but that would require ∼300 subjects. However, when treated as covariates, sex and anthropometrics did not statistically account for the difference in walking economy between runners and walkers. Thus, while it would have been preferable to have a larger sample size with more similar sex and anthropometric matched cohorts, it would not have changed our overall conclusion.

### Future Studies

Based on the results of this study and others, future studies of the effect of age and exercise on walking economy are warranted. Although the average age of our runners and walkers was 69 years, a future study might look to see if running exercise continues to prevent or slow the decline in walking economy in even older runners (over the age of 80 years). It seems plausible that at some age that exercise may not be able to sufficiently offset the normal decline in muscular efficiency and walking economy associated with aging. It is also not known whether there is an intensity threshold of aerobic exercise that is needed to prevent the decline in walking economy. Thus, it would be beneficial for future studies to investigate the relative effect of exercises with different levels of aerobic intensity on walking economy.

### Conclusions

In conclusion, older runners mitigate the age-related deterioration of walking economy. However, older walkers are unable to forestall the decline of walking economy as they require the same metabolic consumption as sedentary older adults. The difference in walking economy between older runners and older walkers remains unexplained due to no substantial differences found in either the kinetic or spatio-temporal data between the groups. Other factors such as decreased muscle co-activation and/or increased muscular efficiency may contribute to the superior walking economy exhibited by the older runners.
